# The Study of Comparative Pathology

**Published:** 1882-01

**Authors:** Thomas E. Satterthwaite


// THE JOURNAL

OF

COMPARATIVE MEDICINE AND SURGERY.

VOL. III. JANUARY, 1882. No. 1.

### ORIGINAL COMMUNICATIONS.

### ART. I.—THE STUDY OF COMPARATIVE PATHOLOGY: INTRODUCTORY LECTURE OF A COURSE, DELIVERED AT THE COLUMBIA VETERINARY COLLEGE, IN 1881–82.

BY THOMAS E. SATTERTHWAITE, M.D.

WE commence, to-day, gentlemen, the study of Comparative Pathology, the precise meaning of which I shall first endeavor to explain to you.

You know that Physiology, as we study it in animals, teaches us about the various activities of the bodily parts, while they are in a healthful condition. But, supposing that we are desirous of studying any form of abnormal or unhealthful activity, it is to Pathology that we turn, as the department of medicine that is calculated to give us the needed information.

Comparative Pathology is merely that broad and comprehensive field, that includes not only the whole range of abnormal animal life, but, I may truly say, the vegetable as well. I should hardly dare to say, that it is my purpose to enter and traverse with you this almost boundless field, but I will say that it is intended, in this course of lectures, to introduce you to that most useful part of it, which includes a combined study and comparison of those morbid manifestations in men and animals that are, in some measure, common to both. This particular department or division of Pathology is also, I may say to you, a somewhat new one, but one which is, nevertheless, destined to assume great importance, so that I have ventured to think it will mark an era, brilliant in novelties, and full of important discoveries, that may elucidate hidden principles of disease, and indicate the direction our efforts are to take in combatting them more successfully than heretofore. To this point reference will again be made.

I think it well, at this time, also, to guard you against the possible error of mistaking Pathological Anatomy for Pathology, as is sometimes done. The former, in its pure sense, is only the substantial framework on which the edifice of Pathology is being reared. It takes cognizance of all the alterations of form, size, shape, color and consistency, as they have been and are observed by the naked eye.

Until the discovery of the compound microscope, a classification founded on such distinctions, was very useful, but with the rise of Pathological Histology, so many new types of disease were recognized, that the entire classification had, in a great measure, to be re-cast. Now, it is upon this standpoint of Microscopic Anatomy that the present scientific position of Pathology chiefly rests. To Physiology it owes less, though it is proper to admit that there are, and always will be, probably, certain classes of abnormal activities not associated with any structural change. It is in such cases that we turn to Physiology, as the only branch of our science that is competent to explain them. True Pathology, therefore, bases itself on these three fundamental studies; indeed, without them it has no proper existence. Its development, accordingly, is to be sought in the gradual growth of these collateral branches.

With the ancients the whole subject was a matter of speculation. It is true that feeble efforts were made, from time to time, to discover the hidden springs of disease, as may be gathered from the writings of Aristotle; but so many restrictions were placed upon the dissection of human bodies, that information was chiefly derived from examining the lower animals. Alcmeon, who flourished in the sixth century (B.C.), and Democritus, who lived in the third and fourth century (B. C.), obtained their knowledge chiefly in this way.

Real progress became possible only during the supremacy of the Alexandrian school, in Egypt, under the Ptolemies, for then systematic post-mortem examinations were allowed and even encouraged. Prominent among the anatomists of this time were Erasistratus, of Ceos (born 300, B. C.) and Herophilus, of the same epoch, while Parthenius, somewhat later (200, B.C.), even published a book, entitled "On the Dissection of the Human Body." With the decline in learning and the associated restrictions again put upon human dissection, anatomy experienced a rapid fall, and Galen (born 131, A. D.) was obliged to conduct his studies on monkeys, or, secretly upon the bodies of persons found murdered, or on such others as came accidentally into his hands.

But a change was destined to take place, and the first substantial signs of it were indicated by Mundinus, of Milan (born 1315). He made public demonstrations of human anatomy, and published a text-book which was used in the Academy at Padua for two hundred years and more. But it was not until the sixteenth century that Pathology began to assert for itself the position it was destined to take at a later day.

It was then that Benevieni, a Florentine physician, wrote a book (1507) entitled "De Abditis Nonnullis Ac mirandis Morborum et Sanationum Causis." With the revival of learning that characterized this century, many important advances were made in anatomy, and Sylvius (Jacques du Bois), appointed professor in the Royal College of France in 1550, and Vesalius, a Flemish physician, of about the same time, published important contributions to human anatomy, laying substantial foundations for subsequent discoveries and demolishing many errors that had been tacitly received and accepted as truth since the time of Galen.

It is common, however, and just, to associate with the name of Morgagni, of Padua, the foundation of Pathology as a science. His well-known work "De Causibus et Sedibus Morborum per Anatomen Indagatis" (1761), translated into many languages, led to his being ranked universally as the most prominent pathologist that had yet appeared, though he was largely inspired by his predecessor, Bonet, who had collected (1675) the records of three thousand post-mortem examinations during a period of two thousand years, and who was himself inspired by Baillou and others. Previous to this, Harvey in his famous manuscript, "De Anatomia Universali" (1616), had indicated the doctrine of the circulation, to the elucidation of which he had undoubtedly been instigated by the zeal of his former master, Fabricius ab Aquapendente.

And yet, in strict justice it should be said that Cæsalpinus (born in 1519) had already described the systematic circulation in his *Speculum Artis Medicœ Hippocraticum*, while Cecco d' Ascoli, also an Italian, had foreshadowed it in the preceding century (1473). Indeed, previous to the birth of Harvey, the pulminary circulation had twice been described, once by Michael Serseties, the Spanish author and physician, in his *Christianism's Restitutio*, completed in 1546, and a second time by Realdus Columbus, a few years later, in *De re Anatomica*, and yet there is abundant reason to presume that each one of the authors reached his conclusions independently; Harvey, who gives credit to Fabricius, being alone excepted.

I should not omit to consider the vast influence at this precise epoch of the great French philosopher and naturalist, René Descartes (b. 1596). One of the founders of modern philosophy and metaphysics, and directing human thought in a way that is difficult to conceive, he still found time to labor in every department of physical science and made contributions to physiology that have entitled him to rank in France with Harvey in England. It was his merit to seek an explanation of all vital processes in simple and universal laws applicable alike to plants, animals, or any mechanism. He taught that we should apply to all operations in the animal frame the ordinary rules of physics. It is needless to say that these fundamental ideas are incorporated into the physiology of to-day.

Borelli, born in Naples in 1608, followed out the pathway thus opened and applied the higher mathematics to medical science. His researches on the actions of muscles and on the bones as levers, have left him an immortal reputation. Incidently he made some pathological contributions of no mean value.

But while pathology was thus prepared to take advantage of a suitable moment to make another prominent step, it remained for Leeuwenhoeck to open up the field in a positive manner. Bending his attention to the making of microscopes, he so increased the magnifying power of the simple convex lens that he was enabled to discover (1671) the red blood globules and spermatozoa, tracing out their characters in various birds, fishes and reptiles. These discoveries were soon extended by his followers, who still further improved upon his lenses and were rewarded by exposing new facts in natural science.

We see, then, how much pathology has had to depend upon the collateral branches of medicine and how true it is in this department of natural science, as in others, that there is a sort of inter-dependence, and one has to wait upon the other, in effecting its own gradual development.

And now, again, the work was taken up by physiology, for to Bichat must be accredited the next great impulse. He died, unfortunately, too early for medical science, leaving an unfinished work on pathology and pathological anatomy, for which he had the greatest taste, and in which he was so successful a teacher, that he has been called "the Morgagni of France." But he accomplished what at that time was more needed—the grounding of some physiological laws that will always remain in force.

To him animal life was the product of separate, and to a certain extent, independent actions. Disease might, therefore, attack certain portions of an organ, but not affect its functional activity to a notable degree. The doctrine of these associated but separate activities in an organ had not been appreciated up to this time.

But you are not surprised to know that the most positive advances in Pathology have been made in very recent times. They are almost wholly connected with the revelations of a single instrument, the compound microscope, that only acquired a proper amount of perfection during the third quarter of the present century. Its teachings, with the associated application of chemical science, have effected a genuine revolution, and established Pathology upon a broad, sound and generally accepted basis. And this is all mainly owing to the influence of a single man, Rudolph Virchow, who lives to-day. He developed the theory that all animal life resides in physiological units (cells), and that to appreciate the actual conditions of disease, we must study them and their relations. This idea permeates all his writings, and is recognized as the modern basis of investigation in all countries. His classifications of disease, taking cognizance, as they do, of these form-elements, are almost universally accepted, and are destined to form a working basis for some time to come. And now, to quote from Huxley, in his address at the last International Congress in London, matters stand thus: "Accurate anatomy has rendered practicable the exploration of the most hidden parts of the organism, and the determination, during life, of morbid changes in them; anatomical and histological post-mortem investigations have supplied physicians with a clear basis upon which to rest the classifi cation of diseases, and with unerring tests of the accuracy or inaccuracy of their diagnosis. If," he continues, "men could be satisfied with pure knowledge, the extreme precision with which, in these days, a sufferer may be told what is happening and what is likely to happen, even in the most distant parts of his bodily frame, it should be as satisfactory to the patient as is is to the scientific pathologist who gives him the information."

It is fortunate for us, perhaps, that the distinguished physicist is not a practicing physician, else we might call him to account for expressions which seem a trifle extravagant; but this much we may fairly say: Many pathological prognoses of the present day have reached a degree of accuracy that is almost mathematical. This is true, more especially, in the domain of neoplasms, or tumors, as you may call them; while, on the other hand, the application of organic chemistry, to the determination of the constituents of suspected fluids and liquids, introduces the precision of the chemical laboratory.

And now we come to another topic, which, after all, is the main one before us, the pathology of diseases in the lower animals, such as the horse, the cow, the dog and the fowl. Now, I have already said that when these topics are taken up and scientifically considered, a new era will be opened for Pathology. Its influence will also be felt in the whole domain of medicine. Indeed, I am not alone in holding this view and making it public.

In a recent address before the Pathological section of the recent International Congress in London, the venerable President insisted that the field of Pathology must be made to embrace all diseases in all animals and in all vegetables. He urged that a comparison between diseases in men and animals may throw much light on their nature, as, indeed, the discussions on hydrophobia, as compared with the rabies, vaccinia compared with variola, have served to elucidate both the human and the animal disease, by calling attention at once to their similarity and distinctiveness. Human Pathology should really have its basis in the comparative, a wealthy mine which has hardly been worked at all. Indeed, Samuel Wilks, in his address, quotes the historian Buckle, who, in his "History of Civilization in England," has said: "The best physiologists distinctly recognize that the basis of their science must include not only the animals below man, but also the entire vegetable kingdom, and that, without this commanding survey of the whole realm of organic nature, we cannot possibly understand even human physiology, still less general physiology. The pathologists," he further states, "are so much in arrear, that the diseases of the lower animals scarcely form part of their plan, while the diseases of plants are almost entirely neglected, although it is certain that, until all these have been studied, and some steps taken to generalize them, every pathological condition will be empirical, on account of the narrowness of the fields from which it is collected."

But it is not to be inferred that Comparative Pathology is a new department altogether, for some scattered work has been done in it.

Among the first, however, who deserve prominence is Jenner, whose experiments on cows led to the introduction of *vaccination*, by which thousands upon thousands of human beings have been saved a fearful and fatal malady, or a life long disfigurement. Hearing from a milkmaid that she felt herself protected against the human disease because she had contracted cow-pox, he pursued the study of this matter in Gloucestershire, and succeeded in finding that there was a peculiar pustular eruption of the cows (there being many eruptions quite similar in appearance) that really protected against small pox. So satisfied did he at length become that the matter was demonstrated, that he experimented upon his son, then a lad less than six years of age.

The advantage of this discovery to the human race may be estimated when we consider that prior to vaccination the mortality in some parts of England was 96 per 1,000, while afterwards it was reduced to less than one-third that amount, and in counties where vaccination became compulsory the mortality actually fell as low as ten and even two per 1,000 souls.

Now, it is singular that physicians never carried out this idea farther, and failed to examine and see whether they could not counteract the virulence of other contagious or infective diseases by inoculating the patient with a virus that would produce the disease in a modified form; and it seems to have been left for the veterinarians, again, to have the honor of having counteracted disease, prolonged life, and diminished the annual losses to the country by experiments in Comparative Pathology.

Before, however, I give the tribute to Toussaint, Pasteur and his follow workers which I believe they fairly merit, let me add that it is perhaps to a physician of this city that we owe the credit of having first made an allied series of experiments, though with a totally different object. These experiments and their practical results were not published, however, until after those of Pasteur, but they are still worthy of recognition. Observing that in certain portions of South America the negroes were prone to die of consumption and a rapid form of it, he conceived the idea of vaccinating them, as it were, with tuberculous matter, thus ensuring them the disease, but this peculiar matter he had first carried through a series of calves, so that the poison was robbed of its excessive virulence. He thus claims to have, by inoculation or vaccination, secured to the negroes an immunity against the fatal form of consumption. This gentleman is Dr. Wesley Miller, of this city. The other topics I shall allude to are those of chicken cholera and splenic fever. Pasteur seems to have found that the ravages of these diseases may be checked by inoculating the animals with a so-called modified virus. Pasteur informs us that he has taken the splenic poison from a chicken, and after introducing it into a vessel, taken a similar amount of fluid from this, and so on with a third until he has diluted the original virus, say 100 times. This fluid then, by inoculation upon healthy chickens, has caused a modified disease which was not fatal, and protected against the original disease. The same thing was done with the splenic fever virus. Now, to prove that he had accomplished this result, fifty sheep were placed at his disposal, and twenty-five were vaccinated. Two weeks afterwards the whole fifty sheep were inoculated with the most virulent anthrax poison. The twenty-five vaccinated sheep resisted the disease, but those that had not been inoculated died of splenic fever within fifty hours. The demand for such treatment accordingly was very great, and in less than twenty days 20,000 sheep and many cattle and horses were inoculated. Pasteur seems to have been antedated in his work by Colin and Toussaint and Burdon-Sanderson, but the work was never done before on so large a scale. It is now an important matter to know how long such inoculation will secure immunity.

It is said that at least five diseases of animals are thus capable of being counteracted by inoculation. But enough of this question at the present time. Let us turn to another disease of the lower animals that is communicated to man. This is, I assure you, gentlemen, a topic that is full of novelties and very little understood by even able and competent human physicians.

Let me read you a letter from Dr. Wesley Miller on this point:

"JULY 27th, 1881.

"DEAR SIR:

"More than forty cases of scarlet fever having appeard at Keswick, in England, the medical officer traced their cause to a dairy from which the infected families received their milk. A single case had been brought to a house next to the dairy. The English are nearer to a successful solution of the problem how to deal with infectious diseases than we, in that almost every town and village has its medical officer, who is obliged to trace the infection to its originating cause; if possible, to isolate all cases of contagious fever; and, no matter what the rank of the patient, to compel the abandonment of the house in which the case has occurred, and its thorough disinfection. In all these actions he has the law to sustain him. With regard to typhoid and scarlet fever, both lately have been repeatedly traced to dairies. Exactly how the poisonous germs are communicated through the milk has not yet been discovered, but of the fact that they are so disseminated through the whole neighborhoods there can be no doubt.

"I am, very respectfully, your humble servant,

"W. MILLER.

"DR. T. E. SATTERTHWAITE,
"50 East Thirty-first street, New York."

Now, if any of you gentlemen were to turn to an excellent and recent publication on Diphtheria, you would find it stated there that "garget" or caked bag in the cow, in some one of its forms, is claimed to have given rise to diphtheria in the human species. This matter has provoked much discussion in England. But the following case furnishes more direct evidence of the way in which the contagion may originate. I take the liberty of introducing these annotations from the above medical book because I am not able to consult the original.*

In the winter of 1875–6, every new-born calf in a certain district of Germany died of a disease that exhibited the following symptoms: More or less abundant salivation, yellow or greenish discharge from the nostrils, swelling of the cheeks, cough and sometimes diarrhœa, ulceration of the cheeks, tongue, palate, nasal fossæ, larynx, bronchi, and intestines, ulcerations about the feet, caseous deposits in the lungs, and commencing pleurisy. Otherwise nothing abnormal. A healthy calf was affected, after being brought in contact with the diseased ones; adult animals not so. But the superintendent

* A Treatise on Diphtheria. By Dr. A. Jacobi. New York, 1880.

of the farm and the woman who attended the calves were taken with angina.

It is well-known that a number of veterinarians regard the epidemic and catarrhal fever of the bovine race as diphtheritic.

Birds are also said to be similarly affected, and mention is made of a parrot that died of tracheal croup, the obstruction resulting from an accumulation of aspergillus, fibrin, and corpuscles. According, also, to Friedberger's report to the Veterinary Society of Munich, there is a croup and diphtheria of the domestic fowls. The thorax, mouth, and larynx, nares, cella-infraocularis, conjunctiva, cornea, sclera, and upper portions of the intestines are affected as in the human subject, while there is a tendency to destruction of the eye. The course of the disease is slow, lasting several months, and the mortality, especially among pigeons, is great.

Nicati studied an epidemic amongst hens, which had similar symptoms to the disease in man then prevailing in the place.

In the *Medical Record*,* of this city, is to be found the notice, that in a house where five children were sick with diphtheria, three kittens took the disease and died. At post-mortem examination, diphtheritic exudations were found in their throats.

These citations are sufficient to show you that there is quite probably a causal rather than a casual relation between these epidemics in mankind and the domestic animals.

You have drawn from what I have said, that the study of human pathology will form part of our scheme. Indeed, man must always be the type for studies in pathological anatomy. It is not only that we have studied his diseased tissues more carefully, but we have a more complete record of the clinical phenomena in his case than can ever be hoped for in comparative medicine. When animals learn to think and talk, we shall be prepared to see them rival man ; until they do so, we never shall have a record of their subjective symptoms ; their hopes and fears, joys and sorrows, all of which, as you readily conceive, enable us always to have a more complete understanding of any particular malady.

Comparative medicine has, it must be said, among its honored names, those who are universally recognized wherever medicine is practised.

Bouley, Chauveau, Davaine and others, who have traced the origin

---

* *Medical Record*, Nov. 8, 1879.

of malignant pustule in man to the carbuncular diseases of animals, have left an impress on the literature of the times. On the other hand, the German veterinarians have enriched human medicine by their researches on trichinosis, the "hydatid" diseases, the "measles of swine," glanders, farcy and hydrophobia, which all come within the proper domain of the veterinary art.

You can realize, therefore, that comparative medicine, whose far-reaching importance I have not even outlined, is not merely broad but affords a greater variety of interesting studies than any other department of medicine.

But in the field of investigation there is still much to be done. You have not only to look anew into pleuro-pneumonia in horned cattle, hog cholera, trichinosis, glanders and hydrophobia, but the whole realm of parasitic disease, whose sway is literally boundless.

So important are these matters now to the country at large, that both the state and general government are having special investigations carried on by paid experts in veterinary medicine. They have expended and should spend adequate sums of money upon them, for they require special training on the part of the investigator, much nicety of manipulation, and the aid of the most modern instruments of scientific precision.

Now you may say to me, How shall we study pathology? I say, first, "make all the post-mortem examinations possible." The mere reading of diseased appearances in books is time thrown away. They can only be appreciated by the eye, which, after taking in the general effect, gradually rejects the immaterial or accidental, and sees underneath the change of color, texture and outline, the really distinctive points that indicate the special lesion.

The great John Hunter, one of the early veterinarians, was a diligent student of comparative anatomy and medicine. This wonderful man, who has been ranked as the "greatest figure in the annals of medicine next to Hippocrates," devoted himself to comparative anatomy during his whole life. He found time to dissect five hundred animals, and even furnished a description of three hundred.

Emulate, therefore, his great example, and never give up the dissection of animals. Such examinations help also to rivet upon your mind the connection between symptoms that have been observed and the morbid states, and in a way that no other method can.

You should also devote yourselves to practical Histology, as I have already indicated. By such a course, only, can you fit yourself to understand the changes that are brought about by disease.

You must realize the full importance of the discovery that tissues are mainly composed of elements called "cells." "The body (I quote from Huxley) is a machine like an army—each cell is a soldier, an organ a brigade, the central nervous system, headquarters and field telegraph, the alimentary and circulatory system, the commissariat. Losses are made good by recruits made in camp, and the life of an individual is a campaign, conducted successfully for a number of years, but with certain defeat in the long run. The efficiency of an army at any given moment depends on the health of the individual soldier and in the perfection of the machinery by which he is led and brought into action at the proper time; and therefore, if the analogy holds good, there can be only two kinds of diseases—the one dependent on abnormal states of the physiological units, i. e., cells; the other, on perturbation or disturbance of their co-ordinating and alimentative machinery."

Still, as stated by Huxley, it is not a study of the cells alone that explains all abnormal phenomena in the body. There are many morbid actions which do not reveal any structural change; hence true Pathology should not maintain that this condition is absolutely necessary.

While, therefore, I recommend you to study the use or uses of the microscope, and assure you that without it you can never be able to understand the scientific medicine of to-day, I do not wish it understood that I think it important for you to study it exhaustively. Any one and all of you can devote such time to it as and ordinary medical student has at his disposal, and he will obtain all the essential points of information that an ordinary practitioner should need to have.

You may ask what I have to say about vivisection. Let me assure you that we, in this country, are never likely to have any hindrance put in the way of our learning what is desirable that we should know from experiments on animals. Fortunately for us physicians, this matter has been taken out of our hands by our national and state authorities, who fully realize the vast importance of scientific experimentation, and will, therefore, protect scientific men in their legitimate pursuits.

Needless cruelty to animals would never, I am sure, be more severely punished than by those who are heartily devoted to the study or practice of medicine.

In conclusion, let me urge upon you to try to retain in your minds all the facts which you have observed. They can never be displaced; but new theories are apt to have short terms of existence. Our text-books and our periodicals are full of them; but of them I say beware. "Prove all things"—that is, turn them over in your mind, and reject them as soon as they fail to stand the tests of logic or experience.

